# Validation of a Method Scope Extension for the Analysis of POPs in Soil and Verification in Organic and Conventional Farms of the Canary Islands

**DOI:** 10.3390/toxics9050101

**Published:** 2021-05-02

**Authors:** Andrea Acosta-Dacal, Cristian Rial-Berriel, Ricardo Díaz-Díaz, María del Mar Bernal-Suárez, Manuel Zumbado, Luis Alberto Henríquez-Hernández, Pablo Alonso-González, Eva Parga-Dans, Octavio P. Luzardo

**Affiliations:** 1 Toxicology Unit, Research Institute of Biomedical and Health Sciences (IUIBS), Universidad de Las Palmas de Gran Canaria, Paseo Blas Cabrera s/n, 35016 Las Palmas, Spain; andrea.acosta@ulpgc.es (A.A.-D.); cristian.rial@ulpgc.es (C.R.-B.); manuel.zumbado@ulpgc.es (M.Z.); luis.henriquez@ulpgc.es (L.A.H.-H.); 2 Department of Environmental Analysis, Technological Institute of the Canary Islands, C/ Los Cactus no 68, Polígono Industrial de Arinaga, Agüimes, 35118 Las Palmas, Spain; rdiaz@itccanarias.org (R.D.-D.); mbernal@itccanarias.org (M.d.M.B.-S.); 3 Spanish Biomedical Research Center in Physiopathology of Obesity and Nutrition (CIBERObn), Instituto de Salud Carlos III, 28029 Madrid, Spain; 4 IPNA-CSIC, Av. Astrofisico Francisco Sánchez, 3, 38206 San Cristóbal de La Laguna, Spain; pabloag10@hotmail.com (P.A.-G.); eva.parga.dans@hotmail.com (E.P.-D.)

**Keywords:** QuEChERS, organochlorine pesticides, PCBs, PAHs, PBDEs, gas chromatography–mass spectrometry

## Abstract

Persistent organic pollutants (POPs) are among the most relevant and dangerous contaminants in soil, from where they can be transferred to crops. Additionally, livestock animals may inadvertently consume relatively high amounts of soil attached to the roots of the vegetables while grazing, leading to indirect exposure to humans. Therefore, periodic monitoring of soils is crucial; thus, simple, robust, and powerful methods are needed. In this study, we have tested and validated an easy QuEChERS-based method for the extraction of 49 POPs (8 PBDEs, 12 OCPs, 11 PAHs, and 18 PCBs) in soils and their analysis by GC-MS/MS. The method was validated in terms of linearity, precision, and accuracy, and a matrix effect study was performed. The limits of detection (LOD) were established between 0.048 and 3.125 ng g^−1^ and the limits of quantification (LOQ) were between 0.5 and 20 ng g^−1^, except for naphthalene (50 ng g^−1^). Then, to verify the applicability of the validated method, we applied it to a series of 81 soil samples from farms dedicated to mixed vegetable cultivation and vineyards in the Canary Islands, both from two modes of production (organic vs. conventional) where residues of OCPs, PCBs, and PAHs were found.

## 1. Introduction

Semi-persistent and persistent organic pollutants (POPs) constitute a large group of compounds that has been widely studied due to their controversial properties. POPs are characterized by their toxicity, resistance to degradation, ability to be transported over long distances, lipophilicity, and tendency to bioaccumulate and biomagnify along the food chain [1]. In this group are compounds of very different nature and usage: industrial applications, i.e., polybrominated diphenyl ethers (PBDEs) and polychlorinated Biphenyls (PCBs); pest control, i.e., organochlorine pesticides (OCPs); or as by-products emitted during the incomplete combustion of organic materials, especially in anthropogenic activities, i.e., polycyclic aromatic hydrocarbons (PAHs) [2,3]. They were used extensively during the 20th century until their restriction, prohibition, or reduction in unintentional emissions following the Stockholm Convention based on their mutagenic, carcinogenic, and endocrine-disrupting properties [4]. Although most of these compounds have been banned in the majority of developing countries for more than 40 years [5,6], many of them still pose a threat to the environment, wildlife, and human health due to their historical use and continuing unintentional emissions.

Soil is regarded as the ultimate sink for persistent organic pollutants, from where they can be emitted to the atmosphere, ground or surface water, and biota [7,8]. POPs can reach soils not only by historical, direct application of OCPs to the crops [9], but also through atmospheric deposition and volatilization processes [10,11]. Thus, even in remote areas where they have never been used, soils can receive inputs of these compounds [11]. The degree to which they are absorbed is influenced by the characteristics of the soil. Clay mineral content, organic matter content, and soil pH can affect their retention, depending on their chemical properties [12]. Given their lipophilic nature, POPs show a high affinity for soil organic matter [13]. Consequently, a part of their input will not be degraded, nor volatilized, nor leached, and may persist for a long time in this medium [14,15]. Although the interaction between the soil matrix and the POPs is stronger, it does not prevent specific plants such as Cucurbitaceae from taking up a part of them [16]. As a consequence, these compounds can find their way into food and feed, leading to bioaccumulation problems in animals and humans [17,18]; thus, biomagnification [19,20].

Humans may be exposed to soil contamination not only indirectly through the ingestion of crops and grazing-animal products, but also by direct contact with soil or even the ingestion of it. The latter might occur through three different pathways: (1) ingestion, (accidental or deliberate, geophagy); (2) inhalation; and (3) dermal absorption or penetration [21]. From these, the ingestion of soil and dust particles may be a potentially important pathway of exposure to environmental pollutants [22]. Accidental ingestion may occur during the consumption of poorly washed fruits and vegetables, through airborne dust, or in hand-to-mouth contact, which is quite common in children [23]. In general, children’s ingestion rates are assumed to be higher than in adults, who may also ingest soil during occupational activities [22]. In addition, unintentional ingestion of soil containing POPs by farm animals raised outdoors may be the main cause of contamination of animal products (meat, milk, or eggs). The most recent studies indicate that soil is a real risk matrix in terms of the transfer of POPs into the food chain [24], especially in areas with high levels of contamination.

For all these reasons, it is relevant to have methods that enable the reliable monitoring of POPs in soils, which is essential to determine not only the level of environmental contamination or the efficacy of remediation measures, but also any potential risk to the population [1].

Traditionally, sample preparation methods used for these compounds in the soil matrix were long and laborious, involved several steps, and employed hazardous and polluting reagents and solvents such as the Soxhlet extraction [25,26], or required expensive equipment such as in microwave-assisted extraction (MAE) [27,28] and pressurized liquid extraction (PLE) [29,30]. To overcome these disadvantages, some authors have used the original or modified QuEChERS method [31,32]. Introduced by Anastassiades et al. in 2003 for the extraction of pesticides from fruits and vegetables [33], it involves an acetonitrile extraction/partitioning step followed by a dispersive solid phase extraction clean-up step. Since then, it has been used for other matrices and analytes due to the high extraction yields using reduce amounts of samples and organic solvents along with its speed, simplicity, and low cost [34]. Once extracted, given that most of them are non-polar compounds, POPs have been analyzed by gas chromatography (GC) coupled to different detectors, particularly electron capture (ECD) [27,35] and mass spectrometry (MS) [36,37]. Nevertheless, due to their highly sensitivity and selectivity, triple quadrupole mass spectrometry (MS/MS) is an ideal technique for both the screening and quantitative analysis of POPs [38,39].

A QuEChERS-based method for the determination of 218 agricultural pesticides in soil has recently been developed in our laboratory. Based on this methodology, we have proposed this scope extension research, with two main objectives: (1) to evaluate and validate a QuEChERS-based and GC-MS/MS method for the quantitative determination of POPs in soils of agricultural origin by GC-MS/MS; and (2) to verify the applicability of this method by analyzing a series of samples of agricultural soils from the Canary Islands, a territory where high levels of POPs have been reported in its population [40,41,42,43] and biota [44,45]. With the validated procedure, we were able to analyze 49 compounds, including 8 PBDEs, 12 OCPs, 11 PAHs, and 18 PCBs, accurately and reliably in 81 soil samples belonging to different agricultural plots of the archipelago.

## 2. Materials and Methods

### 2.1. Reagents, Standard Stock Solutions and Mixes

Analytical-grade acetonitrile (ACN), acetone (Ac) and formic acid (FA, HCOOH) were obtained from Honeywell (Morristown, NJ, USA). AOAC method QuEChERS salts were acquired in commercial premixes from Agilent Technologies (Palo Alto, CA, USA). All the standards of the selected POPs were purchased from CPA Chem (Stara Zagora, Bulgaria). To ensure stability, these compounds were supplied in 5 mixes at 100 μg mL^−1^ each: one for OCPs (in Ac), one for PAHs (in dichloromethane), one for PBDEs (in iso-octane), and two for PCBs (in iso-octane). PCB 200 was used as procedural internal standard (P-IS) and was acquired form Dr. Ehrenstorfer (Augsburg, Germany) at 10 ng μL^−1^. An intermediate solution was prepared by mixing all the commercial standard stocks (20 μg mL^−1^ each compound), and from this, a working solution at 1 μg mL^−1^ in Ac was prepared. Likewise, a working solution of PCB 200 at 1 μg mL^−1^ was prepared in Ac. Matrix-matched calibration curves were prepared by adding the appropriate volume of the standard working solution of standards to blank soil matrix extracts. This matrix extract was obtained by subjecting blank soil samples to the extraction procedure described below. All standard stock solutions, working mix solutions, and matrix-matched calibrators were stored in glass amber vials at −20 °C, and checked periodically for stability.

### 2.2. Sample Collection and Pre-Treatment

The evaluation of the previously developed method [46,47] and its validation following the parameters required for a scope extension [48] was performed using previously selected agricultural soil samples, which did not present any of the analytes of interest. The blank soil samples employed in the validation experiments were considered representative of the most fertile soil of the Canary archipelago (midlands). Samples were taken from a layer between 20 and 30 cm deep during the months of February and September, 2020, and mixed to form a pool. First, soil classification of the samples was carried out using standardized procedures, as previously described [47], and it was found that the samples were of the clay-loam type, with acceptable fertility parameters, containing about 4% oxidizable carbon, and were slightly acidic (pH = 4.88).

For the method performance verification stage, 81 agricultural soil samples were used. All those samples pose similar characteristics to those used for validation. Of these, 35 came from farms dedicated to mixed vegetable cultivation, representative of the most common local agriculture in the Canary Islands (small plots of mixed crops). Nineteen of these farms were dedicated to conventional production (with the use of pesticides), and 16 to organic farming. The rest of the soil samples (n = 46) were collected from vineyards, given the significant growth experienced by the wine sector in the archipelago in recent years (which already has 10 denominations of origin). These samples were collected from farms participating in another study and were evenly distributed between conventionally and organically farmed vineyards (n = 23 of each type). These samples were taken at a depth of approximately 30 cm. All soil samples were homogenized upon arrival at the laboratory and allowed to air-dry at room temperature. Once dried, they were sieved using a 2 mm mesh, and thus considered suitable for analysis.

### 2.3. Sample Preparation

This method is intended as a scope extension of a previously developed method [46,47]; therefore, we did not introduce any variation in the extraction method. Briefly, 10 ± 0.05 g of sieved dry soil were weighted in a 50 mL centrifuge tube. Validation experiment samples and quality controls (QCs) were spiked with the working mixed solution. Next, 50 µL of P-IS solution were added to all samples and blanks. This addition was performed to account for various sources of error throughout all stages of the method [49]. After the spiked process and the P-IS addition, all samples were mixed vigorously and allowed to stand for 1 h before extraction. Then, 10 mL of extraction solution (ACN—2.5% FA) was added and vigorously shaken for 1 min. Next, QuEChERS–AOAC salts [50] (6 g MgSO_4_ and 1.5 g CH_3_COONa) were added and samples were vigorously shaken for another minute. After that, samples were sonicated for 15 min in an ultrasonic bath at 50/60 Hz, 120 V (VWR, Radnor, PA, USA) to facilitate the breakdown of aggregates of clay material and increase the contact of the extractant with soil components. The samples were then shaken in an orbital shaker (Ovan, Barcelona, Spain) for 25 min, and subsequently centrifuged at 3200× *g* for 10 min (Eppendorf 5804 R centrifuge, Eppendorf, Hamburg, Germany). The supernatants were filtered through 0.20 µm (Chromafil^®^ PET, Macherey-Nagel, Düren, Germany) into amber vials and directly analyzed by GC-MS/MS.

### 2.4. GC-MS/MS Analysis

All analyses were performed with a gas chromatograph (Agilent GC 7890B) coupled to a mass spectrometer (Agilent Triple Quad 7010) (Agilent Technologies, Palo Alto, CA, USA). Two 15 m columns (Agilent J&WHP-5MS, 0.25 mm inner diameter and 0.25 µm film thickness each) were used for the separations. The columns were joined together using a purged junction that allowed the use of the backflushing technique (reversal of the carrier gas flow to remove matrix components once all analytes of interest have passed to the second column). The flow rate of the carrier gas (helium, 99.999%) was adjusted whenever necessary by means of the retention time (tR) lock function, using chlorpyrifos methyl (tR = 9.143 min) as a reference. The temperature ramp was programmed as follows: (a) 80 °C—1.8 min; (b) 80 °C to 170 °C at a rate of 40 °C min^−1^; (c) 170 °C to 310 °C at a rate of 10 °C min^−1^; (d) 310 °C for 3 min. The total time for each analysis was 20.75 min. For each analysis, 1.5 µl of the extract were injected in splitless mode. A 4 mm ultra-inert liner with glass wool was used. The injector temperature was programmed at 250 °C. Helium backflushing at 5.8 mL min^−1^ at a temperature of 315 °C for 5 min was used to clean the injector after each analysis.

The MS/MS analyses were performed in multiple reaction monitoring (MRM) mode, for which the spectrometer was programmed in 24 time segments. Depending on the number of MRM transitions in each segment, the cycle time varied within the range 52–334 ms, and the dwell time varied within the range 15–40 ms. The ionization source (electron impact, 70 eV) was maintained at a temperature of 280 °C. Nitrogen gas of the highest purity available (99.9999%, Linde, Dublin, Ireland) was used for Q2 fragmentation of the parent ions at a flow rate of 1.5 mL min^−1^. The transfer line temperature was 280 °C. For data acquisition, a delay of 3.7 min was programmed to allow the solvent front to pass.

### 2.5. Method Validation Parameters

**Figure 1:**
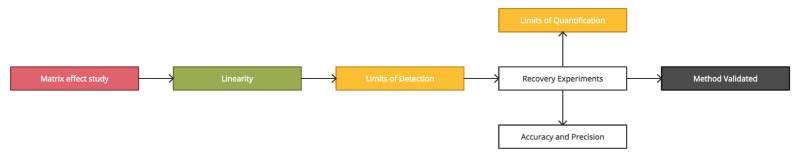
Scheme of the process followed for the method validation.

When extending the analytical scope of a method, it is necessary to perform a validation process to verify the ability of the assay to obtain satisfactory results for new analytes. In this work the validation process included the evaluation of linearity, accuracy, precision, calculation of limits of detection (LOD) and quantification (LOQ), and study of the matrix effect. There is no specific guidance for the validation of methods for the analysis of chemical contaminants in soil; therefore, we decided to follow the European Union guidelines for the analysis of pesticides in agricultural products [49,51]. A diagram summarizing the validation process is shown in Figure 1.

We first studied the linearity of the response by injecting soil extract samples fortified with all analytes at 9 levels (range 0.39–100 ng g^−1^ of soil extract). With the data obtained, the linearity of the curve for each analysis was checked by the Mandel test (ISO 8466-1) using Excel v16.46 (Microsoft Corporation, Washington, DC, USA). To determine the accuracy and precision, the percentage recovery (range 70–120% being acceptable) and percentage relative standard deviation (%RSD, values ≤20% being acceptable), respectively, were calculated. Recovery experiments were performed on blank soil samples fortified at 7 concentrations (in quintuplicates): 0.5, 1.0, 2.5, 5, 10, 20 and 50 ng g^−1^. The lowest concentration level of each analyte that met the criteria for accuracy and precision was considered as the LOQ. On the other hand, the LODs were calculated by calibration approximation [52]. Thus, the lowest level of the calibration curve that had a signal-to-noise ratio (S/N) > 3 and an accuracy between 80–120% was selected. For this purpose, matrix-adjusted calibration curves in the range of 0.024 to 100 ng g^−1^ were prepared in triplicate.

For the confirmation of compound identity, 2 MRM transitions were used: one for quantification (Q), and one for confirmation (q). In relation to the standards in the calibration curve, a maximum deviation of ±30% was tolerated for the ion ratio. In the same way, a maximum deviation of ±0.1 min was established for the retention time.

### 2.6. Statistical Analysis

All statistical analyses were performed with Prism v.3 software (GraphPad Software, San Diego, CA, USA), except for the Mandel test, which was calculated in Excel (Microsoft Corporation, Washington, USA). Precision, both within-runs and between-runs, was calculated using a one-way ANOVA with the number of replicates (usually n = 5) as the grouping variable. The fit to normality of contaminant results in soil samples of the monitoring study was assessed by the Kolmogorov–Smirnov test. This test indicated that, in most cases, the concentrations did not follow a normal distribution, therefore the results, in addition to the mean ± SD, are expressed in terms of median and range. Differences between pairs (conventional vs. organic production farms or mixed vegetable farms vs. vineyards) were tested with the nonparametric Mann–Whitney U test for overall and pairwise comparisons, respectively. For statistical analyses, values below the LOD were assigned a random concentration between 0 and the LOD, and values below the LOQ but above the LOD were assigned a random concentration between these two limits. A *p*-value of less than 0.05 (two-tailed) was considered statistically significant.

## 3. Results and Discussion

### 3.1. Optimization of GC-MS/MS Conditions

First, the GC-MS/MS transitions and chromatographic conditions were optimized by injecting a solution containing the mixture of all the analytes and the P-IS at a concentration of 100 ng g^−1^ each, both prepared in solvent and in the matrix extracted with the procedure employed in the original method [47]. Transitions were selected from those supplied by Agilent Technologies, but prioritizing selectivity over sensitivity due to the complexity of the soil matrix. Subsequently, the collision energies for each transition were optimized by programming sequences of injections from 5 to 60 eV, from which the energy producing the highest intensity response was chosen. Finally, we optimized the dwell and cycle time in a similar way. The compounds are shown by groups and in alphabetical order in Table 1 along with their retention time, transitions, and their collision energies.

**Figure 2:**
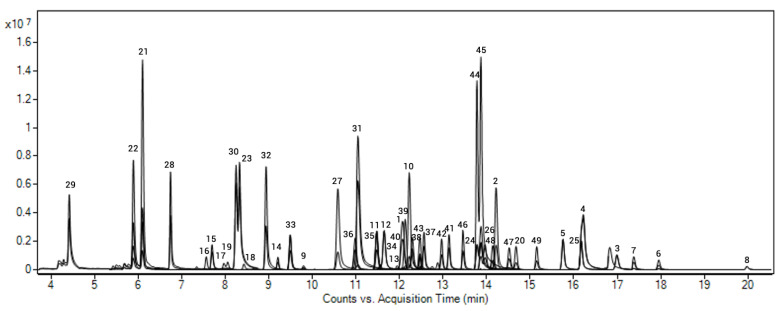
GC-MS/MS chromatogram of a blank soil sample spiked with the selected POPs at 50 ng g^−1^.

We decided to avoid the evaporation and solvent change steps and to inject the final extract as it was obtained (directly into acetonitrile), to avoid the loss of the more volatile compounds. Moreover, ACN has proven to be a good solvent for use in gas chromatography, as stated by Mastovska et al. [53] and from our own studies on pesticides of current or recent use in this matrix [46,47]. No optimization was performed regarding the chromatographic conditions (column and temperature program); being a scope extension, we cannot introduce changes to the original method because we would run the risk of modifying the conditions under which the 218 compounds included in that study were validated. Figure 2 shows the chromatogram of a blank soil sample spiked at 50 ng g^−1^ with the target analytes and P-IS in GC-MS/MS analysis.

### 3.2. Matrix Effect Study

A matrix effect study was run to evaluate the possible interferences of the soil components in the equipment signal. The soil matrix is extremely complex, and its constituents can either enhance or suppress the response, compromising the accuracy, sensitivity, and selectivity of the chromatographic method [54]. In gas chromatography, the presence of high amounts of matrix components could protect the analyte from adsorption or degradation during evaporation in the inlet [55].

We evaluated the matrix effect by comparing the slopes of the calibration curves prepared in the solvent and matrix extracted in the previously detailed conditions according to the equation:

ME (%) = (S_m_/S_s_) × 100 (1)

where ME represents the matrix effect as a percentage, and S_s_ and S_m_ are the slopes of the curve prepared in the solvent and matrix, respectively. The effect of the matrix components on the signal is qualified as the percentage of enhancement or suppression, whether the ME values are above or below 100%, respectively. In accordance to SANCO guidelines, the tolerance range where no significant matrix effects were considered was established as between 80% and 120% [51].

The calibration curves covered the range of 3.125 to 50 ng g^−1^ and were prepared in triplicate, either in the soil matrix or in ACN 2.5%FA, which was the solvent of the final extract. All curves were adjusted to a linear regression curve:

Y = ax + b(2)

where a is the slope and b the intercept. These values were used to calculate ME% using Equation (1).

**Figure 3:**
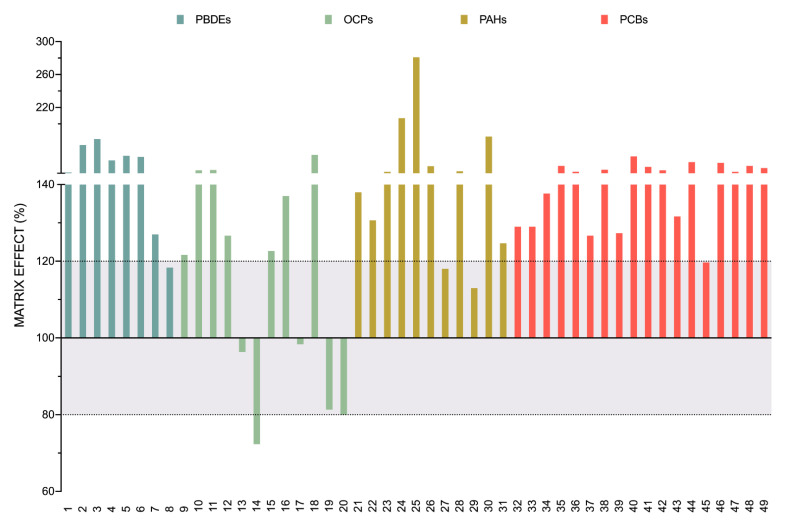
Matrix effect. Bars represent the mean of the ME% of the analytes. Dotted lines represent the tolerance range in which it is considered that no significant matrix effect exists.

Figure 3 shows the ME (%) values for each compound, which are listed following the order assigned in Table 1. Each chemical group has been represented with a different color.

As can be seen, most of the compounds analyzed showed significant matrix effects, except for eight of them (PBDE 183, Endrin, Hexachlorocyclohexane (beta), Hexachlorocyclohexane (gamma-lindane), Mirex, Fluoranthene, Naphthalene, and PCB 157), which were within the limits established for a non-significant matrix effect. The most marked trend was enhancement, although five of the compounds, all of them OCPs, showed no significant to low significant suppression. More than half of the compounds (26 out of 49) had a matrix effect superior to 140%, with Benzo[b]fluoranthene having the highest matrix effect (281%). These data evidence the importance of evaluating the matrix effect in this equipment and the need to use matrix-matched calibration curves in routine analyses of POPs in soil samples.

### 3.3. Method Validation

The method was validated to the extraction and quantitative determination of the above-mentioned POPs under the terms stated in the “Method Validation Parameters” section.

The linearity in the response (R^2^) was superior to 0.99 for all the analytes in the studied range (Table 2). The results of the recovery experiments are presented in Table 3. In terms of accuracy and precision, most compounds met the validation criteria (recoveries between 70% and 120% and RSD < 20%) for concentration between their LOQ and the highest level studied (50 ng g^−1^). The highest level studied was set at 50 ng g^−1^, which is the commonly accepted value for residues in soils [51]. There were some exceptions where recoveries were outside the mentioned range. However, these cases were contemplated in the SANTE guidelines, which accept a recovery between 60% and 140% as long as the RSD is below 20% [49]. Similarly, in some cases, recoveries were within the established limits with RSD slightly higher than 20%, which is considered for residues in soils at concentrations equal to or lower than 10 ng g^−1^ [51].

The LODs were established as between 0.048 and 3.125 ng g^−1^, and the LOQs were between 0.5 and 20 ng g^−1^, except for naphthalene; more than one-half of the analytes had a quantification limit of 1 ng g^−1^ or below (Table 2). For groups, all PCBs and PBDEs had this limit except for PBDE 183, which had a lower limit value (0.5 ng g^−1^); OCPs had a maximum LOQ of 2.5 ng g^−1^; and PAHs had the highest variability in LOQ values, one reaching 50 ng g^−1^ (naphthalene).

The proposed extraction and analytical methods were validated and proved to be reliable and accurate for the analyses of POPs residues in soils.

### 3.4. Application to Agricultural Soil Samples

To verify the performance of the validated method, it was applied to the monitoring of a series of 81 farms in seven of the eight islands of the Canary archipelago (Spain). In 28.4% of the farms (n = 23) no residues were detected, and in the others, the range was between 2 and 10 different residues per farm. In total, we detected 17 different contaminants (4 OCPs, 11 PAHs, and 2 PCBs were detected).

It is striking that no PBDEs were detected in any of the farms, because they are described in the literature as frequent contaminants of agricultural soil [56,57,58]; however, it is true that the reported concentrations are mainly related to the use of treated wastewater [57], or to highly industrialized countries [58]. Previous studies have shown that the rate of flame retardant contamination in the Canary Islands is relatively low [17,18,38], which has been related to the relatively low industrial activity in this region [59,60]. This is probably also the reason why only two PCBs were detected in our samples and at very low concentrations (Table 3 and Table 4), unlike what is reported in agricultural soils from more industrialized regions [61,62], where the reported concentrations are several orders of magnitude higher. Something similar occurred in our series with PAHs; although they were frequently detected, this was mainly due to the high sensitivity of the analytical method, because the concentrations detected were very low (mean ∑PAHs = 2.9 ± 7.2 ng g^−1^, median = 0.0 ng g^−1^). These concentrations are considerably lower than those reported in highly industrialized countries or regions, in the order of 70 [63] to 50,000 times [64] lower.

However, the levels of two contaminants (p,p’-DDE and p,p’-DDD) did attract our attention, not so much because of the frequency of detection, which was similar to that reported in other studies (>70% of the samples), but because of the high concentrations detected. In fact, p,p’-DDE levels in the samples of our series presented mean (364.6 ± 698.7 ng g^−1^) and median (58.7 ng g^−1^) values that are much higher than those reported in other recent studies in Turkey [65], India [66], Poland [67,68], or Iran [69]. In fact, the highest value in our series (2305.6 ng g^−1^), which paradoxically was found in a farm dedicated to organic vegetable farming, was about twice the median value reported for a region of Azerbaijan that has been reported to be historically contaminated by DDT (and its metabolites), because it was subjected to intensive aerial spraying campaigns [70]. It is still surprising that more than 50 years after the banning of DDT, such high levels of its metabolites are still detected. However, as mentioned before, previous studies have indicated that the level of contamination by this insecticide in the Canary Islands has been very high, because historically there has been very intensive usage of this insecticide in agriculture in this archipelago; even today, this region stands out for its intensive use of pesticides, one of the highest rates in Europe [71].

**Figure 4:**
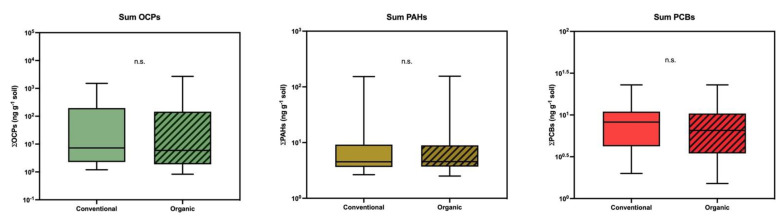
Comparative study of sums of organochlorine pesticides, polycyclic aromatic hydrocarbons, and polychlorinated biphenyls between farms of conventional production and those of organic production. The lines show the medians, the boxes cover the 25th to 75th percentiles, and the minimal and maximal values are shown by the ends of the bars.

It was precisely the fact of detecting such a high level of DDE contamination on an organic farm that led us to extend the sampling and make a comparison between the two types of production. As shown in Table 4, approximately the same types of residues were detected in some farms as in others, and with similar detection frequencies and concentrations. Thus, we did not find statistically significant differences, either in the individual compounds, or in the sums by chemical group (Figure 4). This lack of differences would reflect the still-high levels of residual contamination in this region.

**Figure 5:**
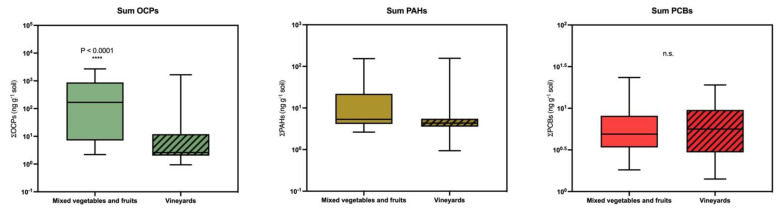
Comparative study of sums of organochlorine pesticides, polycyclic aromatic hydrocarbons, and polychlorinated biphenyls between farms devoted to the production of mixed vegetables and vineyards. The lines show the medians, the boxes cover the 25th to 75th percentiles, and the minimal and maximal values are shown by the ends of the bars.

Finally, we wanted to conduct another comparative study between agricultural soils subjected to a different degree of erosion. The reason lies in the fact that, despite decades since the banning of DDT, gradual declines in its levels were observed in many sites, followed by a second peak of apparent contamination with a new rise in the levels of this insecticide and its metabolites, which could be related to the deposition of new sediments removed from soils subjected to intense erosion [72]. A major cause of agricultural soil erosion is the use of herbicides such as glyphosate and atrazine [72]. In vineyards, the intensive use of herbicides to eliminate weeds between and within vine rows is frequent [73]. This originates from estates with very little vegetation cover, resulting in high soil erosion during rainfall, especially storms, and on hillside estates [74]. Therefore, in this study, we wanted to compare farms dedicated to mixed vegetable crops, with higher vegetation cover and lower herbicide use, with that of vineyards in the Canary Islands. We had not found differences between organic and conventional farms; therefore, we decided not to consider these subgroups. As shown in Table 5 and Figure 5 (summations), we found significant differences in the concentrations of p,p’-DDE, p,p-DDD, and dieldrin, although in the opposite directions to those theorized by other authors [72,73]. Our results indicated that the concentration of organochlorines in farms devoted to mixed vegetable cultivation was much higher than that in vineyards, which would present levels very much in line with those reported recently in studies around the world [65,66,67,68,69]. This further highlights the need for robust and reliable analytical tools to routinely monitor levels of persistent and semi-persistent organic pollutants because, as we have seen, levels can vary greatly from place to place.

## 4. Conclusions

In this study, we have successfully validated a one-step QuEChERS-based method without clean-up for the determination of POPs in agricultural soil samples by GC-MS/MS. It has allowed us to simultaneously extract and analyze 49 POPs (8 PBDEs, 12 OCPs, 11 PAHs, and 18 PCBs). We have proven the applicability of this method in a monitoring study of 81 samples collected in plots dedicated to different purposes in the Canary archipelago and to establish the contamination profile by these compounds. In general, we detected very low levels of industrial contaminants, but very high levels of the main DDT metabolites. This is consistent with the high levels of contamination by these pesticides reported in the past in this archipelago. We found no significant differences in contamination levels between conventional and organic farms. However, we did find that the levels of contamination by organochlorine insecticides in the agricultural soils of this region seem to be strongly associated with the type of crop grown on them; levels were significantly higher in farms dedicated to mixed vegetable crops than in vineyards. Further research should shed light on the presence of DDTs and offer bioremediation solutions in most contaminated soils. The method we have validated has proven to be simple, economical, environmentally friendly, and useful for monitoring studies of POPs in soils compared with previously used methods such as Soxhlet, MAE and PLE.

## Figures and Tables

**Table 1:** Compounds analyzed in soil with their group, retention time, and mass spectrometric conditions.

No.	Compound	Group ^a^	tR (min)	Quantification		Confirmation	
				MRM Transition (*m/z*)	CE (eV)	MRM Transition (*m/z*)	CE (eV)
1	PBDE 28	PBDEs	12.13	406.0 → 246.0	20	406.0 → 167.0	25
2	PBDE 47	PBDEs	14.21	326.0 → 138.0	45	484.0 → 324.0	25
3	PBDE 85	PBDEs	16.99	566.0 → 406.0	25	564.0 → 404.0	25
4	PBDE 99	PBDEs	16.18	566.0 → 406.0	25	564.0 → 404.0	25
5	PBDE 100	PBDEs	15.76	564.0 → 404.0	25	566.0 → 406.0	25
6	PBDE 153	PBDEs	17.96	644.0 → 484.0	30	486.0 → 377.0	30
7	PBDE 154	PBDEs	17.38	644.0 → 484.0	30	486.0 → 377.0	30
8	PBDE 183	PBDEs	19.98	563.6 → 454.7	40	561.6 → 454.7	40
9	Aldrin	OCPs	9.80	263.0 → 228.0	10	255.0 → 220.0	20
10	Dichlorodiphenyldichloroethane (p,p’ DDD)	OCPs	12.22	235.0 → 165.0	20	235.0 → 199.0	30
11	Dichlorodiphenyldichloroethylene (p,p’ DDE)	OCPs	11.48	318.0 → 176.0	60	318.0 → 248.0	15
12	Dieldrin	OCPs	11.58	263.0 → 228.0	15	277.0 → 241.0	15
13	Endrin	OCPs	11.94	263.0 → 193.0	35	245.0 → 173.0	25
14	Heptachlor	OCPs	9.21	272.0 → 237.0	15	274.0 → 239.0	15
15	Hexachlorobenzene (HCB)	OCPs	7.70	284.0 → 214.0	40	284.0 → 249.0	25
16	Hexachlorocyclohexane (alpha, HCH- α)	OCPs	7.56	219.0 → 183.0	10	219.0 → 109.0	10
17	Hexachlorocyclohexano (beta, HCH- β)	OCPs	7.93	219.0 → 183.0	10	219.0 → 109.0	10
18	Hexaclorociclohexano (delta, HCH- δ)	OCPs	8.43	219.0 → 183.0	15	219.0 → 109.0	45
19	Hexachlorocyclohexane (gamma, HCH-**γ,** lindane)	OCPs	8.13	219.0 → 183.0	10	219.0 → 109.0	10
20	Mirex	OCPs	14.68	272.0 → 237.0	10	274.0 → 237.0	10
21	Acenaphthene	PAHs	6.10	153.0 → 152.0	25	153.0 → 151.0	35
22	Acenaphthylene	PAHs	5.89	152.0 → 151.0	25	152.0 → 126.0	30
23	Anthracene	PAHs	8.47	178.0 → 152.0	28	178.0 → 176.0	35
24	Benzo[a]anthracene	PAHs	13.74	228.0 → 226.0	40	228.0 → 202.0	35
25	Benzo[b]fluoranthene	PAHs	16.15	252.0 → 224.0	60	252.0 → 248.0	60
26	Chrysene	PAHs	14.01	228.0 → 226.0	40	228.0 → 227.0	25
27	Fluoranthene	PAHs	10.58	202.0 → 201.0	27	202.0 → 152.0	42
28	Fluorene	PAHs	6.75	165.0 → 163.0	40	165.0 → 139.0	32
29	Naphthalene	PAHs	4.42	128.0 → 102.0	25	128.0 → 127.0	15
30	Phenanthrene	PAHs	8.23	178.0 → 176.0	35	178.0 → 152.0	28
31	Pyrene	PAHs	11.04	202.0 → 200.0	45	202.0 → 201.0	27
32	PCB 28	PCBs	8.94	256.0 → 186.0	25	256.0 → 151.0	50
33	PCB 52	PCBs	9.49	292.0 → 222.0	25	292.0 → 220.0	25
34	PCB 77	PCBs	11.65	292.0 → 222.0	25	292.0 → 220.0	25
35	PCB 81	PCBs	11.47	292.0 → 222.0	25	292.0 → 220.0	25
36	PCB 101	PCBs	10.98	326.0 → 256.0	30	328.0 → 256.0	30
37	PCB 105	PCBs	12.56	326.0 → 256.0	30	328.0 → 256.0	30
38	PCB 114	PCBs	12.29	326.0 → 256.0	30	328.0 → 256.0	30
39	PCB 118	PCBs	12.15	326.0 → 256.0	30	328.0 → 256.0	30
40	PCB 123	PCBs	12.01	326.0 → 256.0	30	328.0 → 256.0	30
41	PCB 126	PCBs	13.14	326.0 → 256.0	30	328.0 → 256.0	30
42	PCB 138	PCBs	12.97	360.0 → 290.0	25	360.0 → 288.0	25
43	PCB 153	PCBs	12.47	360.0 → 290.0	25	360.0 → 288.0	25
44	PCB 156	PCBs	13.86	360.0 → 290.0	25	360.0 → 288.0	25
45	PCB 157	PCBs	13.96	360.0 → 290.0	25	360.0 → 288.0	25
46	PCB 167	PCBs	13.45	360.0 → 290.0	25	360.0 → 288.0	25
47	PCB 169	PCBs	14.52	360.0 → 290.0	25	360.0 → 288.0	25
48	PCB 180	PCBs	14.14	394.0 → 324.0	30	394.0 → 322.0	30
49	PCB 189	PCBs	15.15	394.0 → 324.0	30	394.0 → 322.0	30
	PCB 200	P-IS	14.46	427.8 → 357.8	30	429.8 → 359.8	30

CE—Collision Energy; tR—Retention time. ^a^ PBDE—Polybrominated diphenyl ethers; OCP—Organochlorine pesticides; PAH—Polycyclic aromatic hydrocarbon; PCB—Polychlorinated biphenyl; P-IS—Procedural Internal Standard.

**Table 2:** Linear studies, limits of detection (LOD), and limits of quantification (LOQ) of the POPs.

No.	Compound	Group	R^2^	LOD (ng g^−1^)	LOQ (ng g^−1^)
1	PBDE 28	PBDEs	0.9997	0.390	1.0
2	PBDE 47	PBDEs	0.9984	0.195	1.0
3	PBDE 85	PBDEs	0.9982	0.780	1.0
4	PBDE 99	PBDEs	0.9984	0.390	1.0
5	PBDE 100	PBDEs	0.9966	0.195	1.0
6	PBDE 153	PBDEs	0.9990	0.195	1.0
7	PBDE 154	PBDEs	0.9984	0.390	1.0
8	PBDE 183	PBDEs	0.9981	0.195	0.5
9	Aldrin	OCPs	0.9976	0.390	1.0
10	p,p’ DDD	OCPs	0.9993	1.560	2.5
11	p,p’ DDE	OCPs	0.9975	0.390	2.5
12	Dieldrin	OCPs	0.9951	1.560	5.0
13	Endrin	OCPs	0.9910	1.560	2.5
14	Heptachlor	OCPs	0.9987	0.097	0.5
15	HCB	OCPs	0.9997	0.390	1.0
16	HCH- α	OCPs	0.9948	1.560	2.5
17	HCH- β	OCPs	0.9989	1.560	2.5
18	HCH- δ	OCPs	0.9992	1.560	2.5
19	Lindane	OCPs	0.9993	1.560	5.0
20	Mirex	OCPs	0.9988	0.390	1.0
21	Acenaphthene	PAHs	0.9972	0.780	2.5
22	Acenaphthylene	PAHs	0.9962	0.780	1.0
23	Anthracene	PAHs	0.9993	0.780	5.0
24	Benzo[a]anthracene	PAHs	0.9932	0.780	20.0
25	Benzo[b]fluoranthene	PAHs	0.9967	1.560	10.0
26	Chrysene	PAHs	0.9912	0.780	10.0
27	Fluoranthene	PAHs	0.9963	0.780	20.0
28	Fluorene	PAHs	0.9980	0.390	1.0
29	Naphthalene	PAHs	0.9978	3.125	50.0
30	Phenanthrene	PAHs	0.9991	0.780	5.0
31	Pyrene	PAHs	0.9993	0.780	10.0
32	PCB 28	PCBs	0.9997	0.097	1.0
33	PCB 52	PCBs	0.9986	0.195	1.0
34	PCB 77	PCBs	0.9995	0.195	1.0
35	PCB 81	PCBs	0.9947	0.780	1.0
36	PCB 101	PCBs	0.9995	0.390	1.0
37	PCB 105	PCBs	0.9963	0.097	1.0
38	PCB 114	PCBs	0.9993	0.195	1.0
39	PCB 118	PCBs	0.9968	0.195	1.0
40	PCB 123	PCBs	0.9992	0.097	1.0
41	PCB 126	PCBs	0.9999	0.097	1.0
42	PCB 138	PCBs	0.9996	0.048	1.0
43	PCB 153	PCBs	0.9997	0.390	1.0
44	PCB 156	PCBs	0.9990	0.390	1.0
45	PCB 157	PCBs	0.9978	0.390	1.0
46	PCB 167	PCBs	0.9990	0.390	1.0
47	PCB 169	PCBs	0.9983	0.390	1.0
48	PCB 180	PCBs	0.9943	0.78	1.0
49	PCB 189	PCBs	0.9992	0.195	1.0

**Table 3:** Method validation results: percentage recoveries and relative standard deviations (in brackets).

No.	Compound	Group	0.5 ng g^−1^	1.0 ng g^−1^	2.5 ng g⁻^1^	5.0 ng g⁻^1^	10.0 ng g⁻^1^	20.0 ng g⁻^1^	50.0 ng g⁻^1^
1	PBDE 28	PBDEs	N/A	106.8 (12.3)	116.4 (16.6)	101.1 (6.7)	122.9 (20.7)	105.7 (7.5)	105.0 (6.4)
2	PBDE 47	PBDEs	N/A	98.7 (5.5)	102.7 (9.3)	97.8 (2.9)	114.5 (11.6)	99.3 (4.6)	103.1 (4.8)
3	PBDE 85	PBDEs	N/A	92.5 (7.9)	100.7 (13.7)	88.1 (7.6)	104.1 (11.9)	91.7 (15.7)	99.3 (9.8)
4	PBDE 99	PBDEs	N/A	107.8 (9.7)	107.0 (11.0)	92.3 (10.4)	102.1 (15.4)	96.2 (15.3)	103.1 (9.7)
5	PBDE 100	PBDEs	N/A	97.9 (11.6)	108.4 (10.9)	91.5 (3.5)	112.0 (13.0)	103.6 (6.4)	104.6 (5.9)
6	PBDE 153	PBDEs	N/A	95.0 (9.6)	103.3 (11.2)	86.2 (4.6)	99.4 (15.2)	89.9 (8.9)	96.1 (9.8)
7	PBDE 154	PBDEs	N/A	100.1 (2.4)	99.5 (11.4)	86.6 (6.2)	94.3 (14.2)	87.1 (11.2)	94.2 (7.5)
8	PBDE 183	PBDEs	117.1 (15.3)	80.6 (10.8)	95.3 (8.1)	83.5 (6.3)	96.3 (19.1)	87.3 (9.4)	85.6 (4.8)
9	Aldrin	OCPs	N/A	106.2 (17.0)	112.1 (11.1)	99.9 (13.0)	112.5 (12.1)	100.7 (4.3)	102.2 (5.8)
10	p,p’ DDD	OCPs	N/A	N/A	105.2 (18.8)	89.9 (4.4)	97.5 (17.2)	89.2 (3.1)	86.7 (5.1)
11	p,p’ DDE	OCPs	N/A	N/A	101.3 (13.3)	81.8 (10.8)	107.4 (11.0)	103.5 (6.1)	107.7 (9.9)
12	Dieldrin	OCPs	N/A	N/A	N/A	77.5 (10.0)	116.7 (10.7)	104.0 (4.9)	96.5 (6.2)
13	Endrin	OCPs	N/A	N/A	90.8 (11.1)	77.3 (9.1)	72.1 (12.8)	69.2 (11.5)	67.9 (9.0)
14	Heptachlor	OCPs	99.4 (13.3)	69.8 (13.5)	88.3 (27.1)	74.3 (7.1)	85.4 (21.5)	84.3 (10.4)	82.0 (16.8)
15	HCB	OCPs	N/A	104.2 (8.5)	111.4 (7.0)	102.0 (4.9)	110.0 (14.6)	101.4 (4.3)	103.9 (3.5)
16	HCH- α	OCPs	N/A	N/A	119.6 (7.6)	101.9 (4.4)	100.1 (15.0)	89.3 (10.1)	95.3 (18.2)
17	HCH- β	OCPs	N/A	N/A	99.2 (12.7)	86.3 (9.6)	98.3 (22.7)	99.5 (19.3)	84.6 (7.3)
18	HCH- δ	OCPs	N/A	N/A	81.8 (16.4)	77.7 (5.5)	93.8 (23.1)	89.9 (6.7)	86.6 (7.1)
19	Lindane	OCPs	N/A	N/A	N/A (N/A)	76.9 (15.7)	94.9 (31.6)	94.7 (20.9)	84.1 (5.4)
20	Mirex	OCPs	N/A	82.7 (13.9)	84.5 (19.3)	72.7 (7.5)	83.3 (14.6)	78.4 (7.9)	79.9 (7.3)
21	Acenaphthene	PAHs	N/A	N/A	112.7 (18.6)	117.2 (12.9)	130.4 (19.6)	130.8 (8.9)	122.9 (4.0)
22	Acenaphthylene	PAHs	N/A	117.9 (10.7)	116.8 (8.1)	115.3 (6.3)	118.3 (15.6)	123.1 (12.6)	112.6 (5.7)
23	Anthracene	PAHs	N/A	N/A	N/A	104.8 (8.7)	103.5 (13.9)	101.9 (2.6)	100.9 (8.8)
24	Benzo[a]anthracene	PAHs	N/A	N/A	N/A	N/A	N/A	76.5 (12.1)	86.5 (10.5)
25	Benzo[b]fluoranthene	PAHs	N/A	N/A	N/A	N/A	63.2 (12.7)	67.1 (14.1)	71.7 (9.3)
26	Chrysene	PAHs	N/A	N/A	N/A	N/A	70.1 (19.7)	68.4 (10.0)	76.1 (10.8)
27	Fluoranthene	PAHs	N/A	N/A	N/A	N/A	N/A	109.2 (14.1)	106.9 (9.7)
28	Fluorene	PAHs	N/A	109.4 (17.3)	113.1 (10.3)	121.9 (2.7)	126.8 (19.6)	121.0 (9.9)	122.5 (5.9)
29	Naphthalene	PAHs	N/A	N/A	N/A	N/A	N/A	N/A	116.8 (4.0)
30	Phenanthrene	PAHs	N/A	N/A	N/A	113.8 (13.6)	120.1 (19.1)	120.3 (6.4)	113.3 (7.0)
31	Pyrene	PAHs	N/A	N/A	N/A	N/A	113.4 (19.8)	104.0 (15.5)	102.9 (11.4)
32	PCB 28	PCBs	N/A	102.6 (10.5)	114.5 (9.2)	108.1 (7.3)	120.6 (15.7)	112.7 (5.1)	112.9 (5.4)
33	PCB 52	PCBs	N/A	106.6 (5.8)	119.2 (6.9)	106.9 (4.6)	126.0 (11.8)	110.1 (3.8)	105.5 (5.0)
34	PCB 77	PCBs	N/A	98.3 (4.2)	108.7 (7.4)	106.8 (6.8)	114.2 (11.1)	103.3 (3.5)	98.7 (7.0)
35	PCB 81	PCBs	N/A	107.0 (7.6)	105.6 (4.1)	93.6 (4.2)	106.2 (11.5)	97.3 (5.8)	109.8 (9.6)
36	PCB 101	PCBs	N/A	101.0 (4.9)	111.5 (9.2)	107.6 (8.0)	126.7 (16.1)	111.5 (5.6)	107.5 (6.1)
37	PCB 105	PCBs	N/A	90.6 (8.0)	102.8 (15.6)	99.4 (11.2)	112.2 (11.8)	108.8 (5.1)	110.5 (2.9)
38	PCB 114	PCBs	N/A	107.5 (12.6)	110.6 (10.9)	102.9 (5.4)	117.1 (19.5)	104.1 (6.5)	107.2 (4.5)
39	PCB 118	PCBs	N/A	108.7 (13.9)	109.8 (10.7)	100.8 (8.6)	110.5 (15.4)	97.7 (4.1)	100.4 (20.3)
40	PCB 123	PCBs	N/A	114.8 (9.5)	131.9 (7.3)	128.1 (6.0)	136.5 (13.5)	123.1 (4.9)	111.2 (2.2)
41	PCB 126	PCBs	N/A	102.1 (8.6)	111.0 (9.2)	114.2 (6.3)	115.4 (13.9)	98.9 (6.2)	91.7 (5.8)
42	PCB 138	PCBs	N/A	92.3 (10.0)	108.7 (17.7)	111.7 (7.7)	120.3 (22.5)	88.6 (12.7)	81.5 (8.3)
43	PCB 153	PCBs	N/A	103.6 (15.3)	109.2 (5.6)	110.0 (6.2)	121.6 (15.9)	105.5 (4.3)	104.5 (3.2)
44	PCB 156	PCBs	N/A	102.0 (6.1)	110.6 (3.5)	107.1 (10.1)	109.2 (9.9)	98.1 (10.5)	88.5 (6.9)
45	PCB 157	PCBs	N/A	103.5 (12.7)	102.5 (9.1)	97.0 (6.0)	103.3 (19.1)	95.5 (5.6)	103.3 (14.5)
46	PCB 167	PCBs	N/A	102.1 (9.9)	106.5 (10.7)	101.9 (5.4)	110.9 (16.1)	101.3 (4.7)	105.3 (7.6)
47	PCB 169	PCBs	N/A	108.2 (8.5)	100.9 (8.7)	96.1 (4.5)	106.4 (12.5)	93.7 (4.4)	101.1 (6.5)
48	PCB 180	PCBs	N/A	104.5 (6.4)	100.0 (16.6)	97.3 (5.6)	102.0 (10.5)	87.3 (3.9)	99.0 (10.1)
49	PCB 189	PCBs	N/A	104.9 (8.6)	114.9 (2.3)	103.7 (2.1)	118.4 (12.6)	103.9 (11.5)	95.9 (6.5)

**Table 4:** Comparative study of the POPs detected in 44 samples of agricultural soil of farms of conventional production and 37 samples from farms of organic production. Concentrations are expressed in μg kg^−1^.

	Conventional Production				Organic Production				
Compound	Mean ± SD	Median	P25–P75	Freq. (%)	Mean ± SD	Median	P25–P75	Freq. (%)	*p*-Value
DDE	170.6 ± 309.9	5.3	0.3–156.9	68.2	295.0 ± 697.7	3.0	0.3–108.4	65.8	0.7549
DDD	31.9 ± 54.4	1.4	0.5–38.1	45.6	39.2 ± 96.5	1.2	0.7–18.6	39.5	0.5156
Dieldrin	5.1 ± 18.0	1.1	0.4–1.4	13.6	21.5 ± 59.9	0.8	0.3–3.1	23.7	0.9834
Lindane	-	-	-	0	0.4 ± 0.9	0.3	0.2–0.5	2.6	0.4574
Acenaphthene	0.7 ± 1.5	0.4	0.2–0.6	4.5	0.5 ± 0.3	0.4	0.2–0.6	7.9	0.9539
Acenaphthylene	-	-	-	0	0.4 ± 0.3	0.3	0.1–0.5	7.9	0.1224
Anthracene	1.0 ± 2.5	0.5	0.1–0.7	4.5	0.4 ± 0.7	0.3	0.1–0.6	2.6	0.2252
Benzo[a]anthracene	1.1 ± 1.7	0.4	0.2 – 0.7	15.9	1.8 ± 4.7	0.4	0.1–0.7	13.2	0.9318
Benzo[b]fluoranthene	0.6 ± 1.5	0.3	0.2–0.5	4.5	3.9 ± 12.1	0.4	0.2–0.7	7.9	0.0975
Chrysene	0.4 ± 0.8	0.3	0.1–0.6	2.3	1.8 ± 5.3	0.4	0.3–0.5	2.6	0.0591
Fluoranthene	4.3 ± 9.5	0.4	0.2–0.7	22.7	2.2 ± 4.9	0.5	0.2–0.7	15.8	0.7478
Fluorene	0.7 ± 1.6	0.4	0.2–0.6	4.5	-	-	-	0	0.8369
Naphthalene	0.6 ± 1.0	0.4	0.2–0.6	4.5	-	-	-	0	0.4631
Phenanthrene	3.8 ± 11.5	0.6	0.3–1.4	27.3	1.3 ± 2.2	0.4	0.2–1.3	26.3	0.5155
Pyrene	3.6 ± 7.4	0.4	0.2–1.6	25.0	2.2 ± 5.1	0.4	0.2–0.6	15.8	0.5740
PCB28	0.4 ± 0.2	0.3	0.2–0.6	11.4	0.4 ± 0.2	0.4	0.2–0.6	21.1	0.9650
PCB52	0.5 ± 0.2	0.5	0.3–0.7	4.5	0.4 ± 0.2	0.5	0.2–0.6	7.9	0.6091

**Table 5:** Comparative study of the POPs detected in 35 samples of agricultural soil of farms devoted to mixed vegetables and fruits and 46 samples from vineyards. Concentrations are expressed in μg kg^−1^.

	Conventional Production				Organic Production				
Compound	Mean ± SD	Median	P25–P75	Freq. (%)	Mean ± SD	Median	P25–P75	Freq. (%)	*p*-Value
DDE	456.2 ± 721.6	148.8 ****	5.8–568.9	91.4	58.4 ± 185.3	0.5	0.2–7.9	51.0	<0.0001
DDD	55.8 ± 66.5	32.6 ***	0.9–119.0	62.8	19.2 ± 78.8	1.2	0.5–2.7	27.7	0.0006
Dieldrin	27.3 ± 63.8	1.3 *	0.4–5.6	34.3	1.8 ± 5.0	0.8	0.5–1.3	6.4	0.0240
Lindane	-	-	-	0	0.4 ± 0.9	0.3	0.1–0.5	4.3	0.9478
Acenaphthene	0.9 ± 1.7	0.5	0.3–0.7	14.3	-	-	-	0	0.0964
Acenaphthylene	0.5 ± 0.3	0.4	0.3–0.6	8.5	-	-	-	0	0.3506
Anthracene	1.1 ± 2.7	0.4	0.2–0.6	5.7	0.5 ± 0.6	0.5	0.1–0.7	2.1	0.7503
Benzo[a]anthracene	1.3 ± 1.8	0.6	0.3–1.5	25.7	1.5 ± 4.2	0.5	0.2–0.7	8.5	0.4825
Benzo[b]fluoranthene	-	-	-	0	3.4 ± 10.8	0.5	0.2–0.7	17.0	0.2092
Chrysene	-	-	-	0	1.6 ± 4.7	0.4	0.2–0.6	8.5	0.8092
Fluoranthene	5.7 ± 10.4	0.6	0.3–6.6	31.4	1.6 ± 4.3	0.5	0.2–0.7	10.6	0.2515
Fluorene	0.8 ± 1.8	0.4	0.2–0.6	5.7	-	-	-	0	0.9766
Naphthalene	0.6 ± 1.1	0.4	0.1–0.6	5.7	-	-	-	0	0.5803
Phenanthrene	4.9 ± 12.8	0.6	0.5–4.2	40.0	1.0 ± 1.9	0.5	0.3–0.7	17.0	0.0315
Pyrene	4.8 ± 8.2	0.4	0.2–7.6	31.4	1.5 ± 4.3	0.3	0.2–0.8	14.9	0.1583
PCB28	-	-	-	0	0.4 ± 0.3	0.3	0.2–0.6	27.7	0.4995
PCB52	0.4 ± 0.2	0.4	0.1–0.6	14.3	-	-	-	0	0.6052

*— *p*< 0.05; ***— *p* < 0.005; ****— *p* < 0.001

## Data Availability

The data presented in this study are available on request from the corresponding author.
